# Supplementary material for: Beyond Pain Management: Skin-to-Skin Contact as a Humanization Strategy in Cesarean Delivery: A Randomized Controlled Trial
Source: Healthcare (Basel). 2025 Jul 30;13(15):1866. doi: 10.3390/healthcare13151866 (PMC12346837; doi:10.3390/healthcare13151866)
Supplement: Supplementary file 1 [file healthcare-13-01866-s001.zip › healthcare-3713720-supplementary.pdf]

CARE CIRCUIT FOR CAESAREAN SECTION AND MOTHER-BABY SKIN-TO-SKIN CONTACT

JUSTIFICATION:

In the line of good practices in "Humanised care of the birth process", continuity of care and quality of care, to guarantee the safety of the mother and baby as well as the suitability of the physical environment, the following circuit of care for pregnant women after caesarean section is established, favouring skin-to-skin contact between mother and baby.

GENERAL OBJECTIVE:

To increase the level of satisfaction perceived by the pregnant woman and families in relation to the quality of care received, in the context of the humanisation of birth.

SPECIFIC OBJECTIVES

1) To guarantee mother-baby safety.
2) To increase the quality of care provided and perceived.
3) To promote multidisciplinary teamwork in the care of pregnant women during the birth process, favouring continuity of care in the different stages of the process.

CARE CIRCUIT FOR PREGNANT WOMEN:

The pregnant woman with the need to terminate her gestation through a caesarean section will go to the operating theatre in bed, accompanied by her referring midwife and the unit's orderly.

PROFESSIONALS INVOLVED:

Unit orderly.
Midwife and midwife of the antepartum unit.
Anaesthetist on duty

DOCUMENTATION:

- Patient identification tag.
- Maternal and newborn identification bracelet.
- Signed informed consent forms.
- Identification document for the newborn.
- Paediatric History.
- Digital history: Obstetric sheet of the pregnant woman.
- Post-surgical anaesthesia sheet.

PROCEDURE.

Main functions of the different professionals in the caesarean section assistance circuit.

Unit supervisor:

- * Transfer the pregnant woman with indication for Caesarean section, in bed, to the operating room for this purpose, placing the woman on the operating table.
- Request a bed in the general admission or emergency department for her subsequent admission to the hospital ward.
- * Transfer the mother with her NB in CPP from the operating theatre to the antepartum room where post-surgical care will be provided.
- * Transferring the mother-in-waiting with her NB in CPP from antepartum 8 to the assigned room in the hospital ward.

Prepartum referral TCAE:

Before moving to the surgical area:

- If necessary, shave the pubic area of the pregnant woman before going to the operating theatre.
- Check the removal of any metallic or mobile object that could put the pregnant woman at risk.
- Accompany and install the family member in postpartum 1, previously leaving their belongings in antepartum 8 where the established post-surgical care will be carried out.

In the surgical area:

- Making the mother-child identification bracelets.
- Collaborate in all the functions of their category that are necessary during the paediatric assessment of the newborn.

In antepartum 8:

- She will take an interest in the patient's condition by observing and assessing the patient's needs together with the midwife, taking an active role. Bed hygiene should be offered to the patient.
- Control of the amount of blood in the gynaecological compresses that are changed on the patient.
- Checking diuresis. If necessary, check hourly diuresis.
- Calls/ringers from the patient will be attended to by both the TCAE and the midwife.
- The midwife will make sure that the mother is comfortable, and will come whenever she requests her presence.
- How the arranged caesarean section is transferred to the ward.
- Collaborate in all the functions of their category that are necessary.
- The room will be clean, tidy and ready for its next use.

MATRONA (midwife in charge of pre-birth):

- Prepare the newborn's resuscitation cot with all the necessary material, and inform the paediatrician by telephone of the reason for the caesarean section.
- Receive the newborn in a sterile drape and place him/her on the neonatal resuscitation cot for paediatric assessment.
- Assist the paediatrician in all duties related to his/her category.
- After paediatric assessment, weigh the newborn, clamp the umbilical cord, identify the newborn and prepare the newborn for skin-to-skin contact with the mother on the operating table as soon as possible and if the condition of both mother and newborn permits.
- If the condition of the mother and the newborn permits, maintain skin-to-skin contact throughout the surgical procedure.

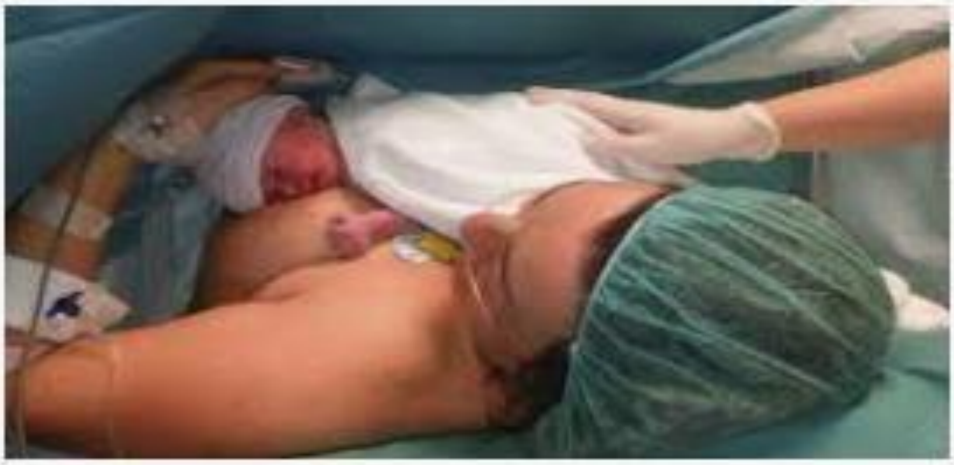

- On arrival of the expectant mother at antepartum 8, the mother will be placed on the multifunctional monitor to monitor the established vital signs.

The patient should remain under close surveillance and continuous monitoring for a minimum of 90-120 min. Clinical monitoring is important and should be performed repeatedly to detect the onset of complications early.

Clinical monitoring shall be based on:

- **Sensory/motor block**: An absence of motor block according to the Bromage scale and an absence of sensory block (recovery of sensation, pain, pressure, touch and temperature) should be confirmed.
- **Uterine tone and height**: The tone of the uterus and its height in relation to the level of the umbilicus should be assessed repeatedly during admission.
- **Assessment of bleeding**: Vaginal bleeding and bleeding from the surgical wound, staining of dressings or collection in drains (if any) and gynaecological compresses throughout your stay will be quantified.
- **Diuresis**: The volume of diuresis will be measured. On certain occasions a urinometer will be used to quantify hourly urine output.

Monitoring during admission:

- The midwife will be responsible for monitoring and surveillance of the patient during immediate post-surgical care until her subsequent transfer to the hospital ward.
- The referring anaesthesiologist will be the epidural pager bearer, who will be notified in case of any doubt or event. He/she will be responsible for authorising the transfer of the patient to the hospital ward after the 10/10 Aldrete test.

As these are patients with low-risk pathology, non-invasive monitoring is recommended:

- * 3/5-lead electrocardiogram: heart rate, sinus rhythm, unknown rhythm disturbances, ST disturbances....
- * Pulse oximetry saturation: avoid desaturation, maintain SpO2> 94-96%, if a lower value is recorded, administer supplementary oxygen therapy through nasal goggles at 2.5-3 lpm and notify the anaesthesiologist.
- * Non-invasive blood pressure: minimum every 15 min.

Monitoring will be maintained throughout your stay and will be removed prior to discharge. discharge.

- During the entire immediate post-surgical care, the expectant mother will be kept on an absolute diet, starting oral tolerance when the anaesthesiologist indicates this in the patient's clinical history.

- Before being transferred to the hospital ward, the midwife will remove the epidural catheter by medical indication.

MANAGEMENT OF POST-OPERATIVE PAIN.
NUMERICAL OR VISUAL ANALOGUE RATING SCALE . PROTOCOL FOR ANALGESIC TREATMENT

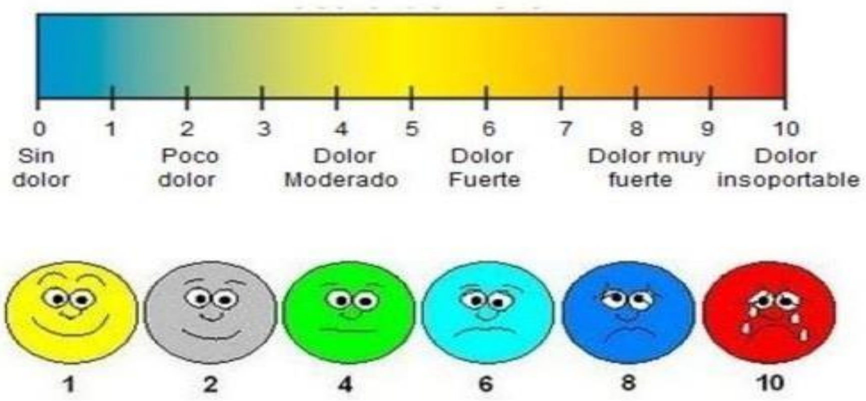

SKIN-TO-SKIN AFTER CAESAREAN SECTION

CPP consists of placing the NB naked, early after birth, in prone position, directly on the mother's chest and abdomen, free of clothing. This practice is recommended by numerous scientific societies for both vaginal and caesarean births, given the numerous benefits for both mother and newborn.

Current recommendations speak of up to 120 minutes in order to benefit mother and newborn with all its advantages, thus taking advantage of the newborn's first sensitive period.

The midwife in charge should provide the following information and make sure that mother and attendant understand it:

- Accompaniment should be permanent.
- They should observe the baby's activity and colour, and prevent obstruction of the nose. Parents should know that any irregular breathing or change in colour should be reported promptly.
- If the mother is very tired or sleepy, the attendant should be advised to watch the newborn, or even hold the baby for skin-to-skin contact. If there is no attendant nearby and the mother is very tired or sleepy, the baby should not be placed in prone position on the mother's abdomen or chest, but in a cot and in supine position.

- The recommended position for the mother is semi-recumbent at 30-45°, with a pillow under her head. Apparently, in this maternal position, it is more difficult for airway obstruction to occur.

- The use of mobile phones during CPP poses a danger to the NB, as it reduces attention to the baby. Discontinuation of CPP should be considered if the mother or companion is distracted despite being informed.
